# Human Papillomavirus type distribution in invasive cervical cancer in Uganda

**DOI:** 10.1186/1471-2334-8-85

**Published:** 2008-06-24

**Authors:** Michael Odida, Silvia de Sanjosé, Wim Quint, Xavier F Bosch, Joellen Klaustermeier, Elisabete Weiderpass

**Affiliations:** 1 Department of Pathology, Faculty of Medicine, Makerere University, Kampala, Uganda; 2 Department of Medical Epidemiology and Biostatistics, Karolinska Institutet, Stockholm, Sweden; 3 Unit of Infections and Cancer, Catalan Institute of Oncology, Barcelona, Spain; 4 DDL Diagnostic Laboratory, Voorburg, The Netherlands; 5 Department of Etiological Research, The Cancer Registry of Norway, Oslo, and Department of Community Medicine, Tromso University, Norway; 6 Department of Genetic Epidemiology, Samfunded Folkhalsan, Helsinki, Finland

## Abstract

**Background:**

We conducted a study aiming to describe Human Papillomavirus (HPV) type distribution in invasive cervical carcinoma in Uganda.

**Methods:**

191 archival cervical carcinoma samples diagnosed in the Department of Pathology, Makerere University in Kampala between 1968 and 1992 were analysed using a sensitive PCR-Reverse Hybridization Line Probe Assay.

**Results:**

Out of the 186 cases of confirmed invasive cervical cancer in the study paraffin blocks, 114 were positive for HPV DNA. Specific HPV genotypes were identifiable in 109 cases: HPV 16, 18, 31, 35, 39, 44, 45, 51, 52 and 70. These occurred as single infections in 105 cases (96.3%) and as multiple infections in 4 cases (3.7%). HPV 16 or 18 accounted for 80% (84/105) of cases with single infection.

**Conclusion:**

The results of this study confirm the role of HPV 16 and 18 in cervical cancer pathogenesis in the Ugandan population. The results suggest that the currently available HPV vaccines against HPV 16 and 18 could possibly prevent the majority of invasive cervical cancers in Uganda.

## Background

Clinical and epidemiological studies have clearly established that infections by certain human papillomaviruses (HPVs) types are causally linked to cervical cancer development [1-5]. Among the high risk HPV types, HPV 16 and 18 are recognized as the main causes of invasive cervical cancer and its precursor lesions [3]. These two viral types were found in most cases of invasive cervical cancer from 22 countries around the world [4]. HPV 16 tends to predominate in squamous cell carcinomas whereas HPV 18 often predominate in adenocarcinomas [4,6-8]. Some studies have also shown that there are geographical variations in HPV type distribution [1,4,9,10]. Finally, recent data shows that HPV immunization offers the greatest possibility for prevention of cervical cancer and has become an important health priority to many governments and health organizations worldwide. HPV vaccines that are currently available include VLPs for HPV 16 and 18 [11,12] as well as HPV 11 and 6 [13]. However, geographical variations in HPV type distribution might influence the currently available HPV vaccine efficacy. Compared to other continents (such as South America and Asia), there is relatively little information about HPV types in invasive cervical cancer in Africa. We conducted a study aiming to characterize HPV type spectrum in the archival specimens from cervical cancer cases from Uganda population including cases diagnosed over 34 years.

## Methods

191 samples from patients with histological diagnosis of invasive cervical carcinoma from 1968 to 1992 were obtained from the archives of the Department of Pathology, Makerere University in Kampala, Uganda. These samples accounted for less than 5% of cervical carcinomas diagnosed during the study period. Nine cases (4.7%) were from the period of 1968 to 1969, 66 (34.6%) cases from 1970–1979, 74 (38.7%) from 1980–1989 and 42 (22.0%) were from the period 1990–1992. Until the early nineties, the Department of Pathology received most specimens for histopathology services from all hospitals and health facilities in the country.

Samples were received in formalin, which may not always have been buffered. Tissue specimens were usually processed within 24 hours of their arrival to the hospital, but for a fraction of samples transported from remote parts of the country, longer times may have been required. Samples were selected randomly from the register of Pathology Department starting in 1968; we oversampled of adenocarcinomas (1:1) because of the special interest to compare genotype distribution in the two major histologies of cervical cancer.

When at the testing laboratory, the paraffin embedded tissue blocks were re-embedded in fresh paraffin wax and four sections (sandwich method) were cut for testing under strict conditions to avoid potential contamination. The first and the last sections were stained with Haematoxylin and Eosin (H&E) to confirm diagnosis and to ascertain the suitability of the tissue to continue for HPV testing. The sections in-between were collected in a screw-top Eppendorf tube for HPV testing. A tissue-free paraffin block was cut after each study block to avoid any HPV carry-over from block to block. A new blade was used for each block and the microtome was cleaned with a vacuum cleaner, Histoclear II and 70% alcohol. To further control for possible sources of contamination, paraffin blocks containing non HPV related lesions (appendicitis, lung tumour, ovary, lymph node etc.) were included blindly in the process at a ratio of 5%, and blank paraffin sections were simultaneously tested.

Proteinase K (Sigma) digestion for 16 hours at 56°C temperature was used to obtain a tissue lysate containing DNA from the paraffin inner 5 μm sections. SPF10 PCR was performed using 10 μl of a 1:10 dilution with water of the tissue digest in a final reaction volume of 50 μl [14]. The amplified PCR products were tested using a probe hybridization with a cocktail of conservative probes recognizing at least 54 mucosal HPV genotypes in a microtitre plate format for the detection of HPV DNA. Optical densities (OD_450_) were read on a microtitre plate reader. HPV DNA positive samples were subsequently analysed by HPV SPF10-LIPA_25 _(version 1: produced at Labo Biomedical Products, Rijswijk, The Netherlands) [14], a reverse hybridization technique that detects 25 high-risk and low-risk HPV types (6, 11, 16, 18, 31, 33, 34, 35, 39, 40, 42, 43, 44, 45, 51, 52, 53, 54, 56, 58, 59, 66, 68/73, 70, 74). The sequence variation within the SPF10 primers allows the recognition of these different HPV genotypes, except for the types 68 and 73 as their interprimer regions are identical and cannot be distinguished on this test. After PCR, 10 μl of the amplimers was used to perform reverse hybridization for HPV genotype identification. The positive hybridization on the strips is visualized as a purple band by means of a precipitating colour substrate on the probe site. Specimens that were HPV/DNA positive but did not hybridize with any of the 25 probes were coded as HPV type X (unknown type). Amplification of a fragment of the human β-globin gene was performed in all HPV/DNA negative samples to assess DNA quality. HPV type-specific distributions were calculated among HPV positive women. All SPF10-LIPA25 PCR detection and typing was performed at the facilities of DDL Diagnostic Laboratories (DDL, Voorburg, Netherlands) and at Institut Català d'Oncologia (ICO, Barcelona). HPV detection was evaluated by histology classification (adenocarcinoma vs. squamous cell carcinoma), year of diagnosis and presence of necrosis in the tissue.

Ethical approval for the reported study was obtained from Ethical Committee at Makerere University, Kampala, Uganda.

## Results

Of the 191 paraffin blocks corresponding each one to a cervical cancer cases, 186 harboured invasive cervical carcinoma cells and, according to protocol, were considered suitable for HPV detection. Five were excluded due to extended necroses seen on the histological slide. 146 were diagnosed as squamous carcinomas, 35 were adenocarcinomas, 3 adenosquamous and two were undifferentiated carcinomas. Out of these, 114 were HPV positive and in 72 HPV could not be detected. All the negative samples tested negative for the presence of β-globin gene indicating a poor quality tissue. Table 1 shows the overall β-globin gene and HPV positivity results according to histological types. There was no statistical differences between histological type and overall HPV infection (Chi2 = 3.64, p = 0.3). There was stability of genotype distribution of HPV 16 and HPV 18 over time, taking into account histology.

**Table 1:** HPV/DNA detection by cervical cancer histological types

	**HPV negative**^a^	**HPV positive**^b^	**Total**
Squamous carcinoma	54	92	146
Adenocarcinoma	15	20	35
Adenosquamous	1	2	3
Undifferentiated carcinoma	2	0	2

**Total**	72	114	186

^a ^HPV not detected. All these samples were human β-globin gene negative, and therefore inadequate for HPV determination

^b ^All β-globin gene positive

Specific HPV types were identifiable in 109 cases, while in the remaining five cases no specific HPV types could be identified (Type X). Ten specific HPV types: 16, 18, 31, 35, 39, 44, 45, 51, 52 and 70 were identified. These occurred as single infections in 105 cases, double infections in three cases and triple infections in one case. Analysis of the HPV genotypes distribution among the HPV positive cases showed that HPV 16 and 18 were the most frequent types, followed in descending order by HPV 45, 31, 35, 51, 39 and 52. Table 2 shows the distribution of HPV infections and histological types.

**Table 2:** HPV type distribution according to histological types

**HPV types**	**Squamous carcinomas**	**Adenocarcinomas**	**Total**
	**Infection with single HPV**		110 (96,5%)

	N (%)	N (%)	N (%)

HPV 16	44 (47.8)	7 (35.0)	51 (44.7)
HPV 18	21 (22.8)	10 (50.0)	33 (28.8)^a^
HPV 16 or 18	65 (70.6)	17 (85.0)	84 (73.7)^a^
HPV 31	4 (4.3)	0	4 (3.5)
HPV 35	2 (2.2)	0	2 (1.8)
HPV 39	1 (1.1)	0	1 (0.9)
HPV 45	10 (10.9)	1 (5.0)	11 (9.6)
HPV 51	1 (1.1)	1 (5.0)	2 (1.8)
HPV 52	1 (1.1)	0	1 (0.9)
Type X	4 (4.3)	1 (5)	5 (4.4)

	**Infection with multiple HPV**		4 (3.5%)

HPV 16/45	1 (1.1)	0	1 (0.9)
HPV 16/51	1 (1.1)	0	1 (0.9)
HPV 45/70	1 (1.1)	0	1 (0.9)
HPV 16/44/52	1 (1.1)	0	1 (0.9)

**Total**	92	20	114^a^

^a ^Includes two additional cases of adenosquamous carcinomas.

In single infections, type 16 occurred in 51 of the cases, type 18 in 33 cases, type 45 in 11 cases, type 31 in four cases, type 35 and 51 in two cases, types 39 and 52 each in one case. The three double infections were found to be combinations of types 16/45, 16/51 and 45/70 while the triple infections included the HPV types16/44/52. When the analysis was restricted to the histological types of cervical carcinoma, HPV 16 was the most frequent in squamous cell carcinomas (47.8% of cases) while in adenocarcinomas, HPV 18 was the most prevalent type (50%). Of the squamous cell carcinomas, 44 of the cases presented with HPV type 16, 21 of the cases with type 18, 10 with type 45, four with type 31, two with type 35, three single infections with types 39, 51, and 52, three double infections and one triple infection. In the adenocarcinomas, 10 were found to have HPV type 18, 7 type 16, one type 45, and one type 51. Concurrent CIN lesions were seen in 18 cases with single HPV infections and one case with multiple HPV types. No differences in HPV detection were observed between the cases with and without pre-neoplastic lesions (Table 3).

**Table 3:** Multiple HPV genotypes in invasive cervical carcinoma cases with and without concurrent CIN lesions

	**HPV + (1 type)**	**HPV + (>1 type)**	**Total**
No CIN	87 (96.7%)	3 (3.3%)	90
With CIN 1/2/3	18 (94.7%)	1 (5.3%)	19

**Total**	105	4	109

p > 0.05

## Discussion

This study shows that HPV/DNA was detected in 114 out of 186 invasive cervical cancer samples, giving an overall HPV prevalence of 61.3% of the samples. If the β-globin gene results are taken into account, then HPV was detected in 100% of the samples from which cellular DNA was obtained.

Specific HPV genotypes were identified in 109 cases using the highly sensitive SPF10 LiPA technology. Most of the infections were due to single HPV types with only four cases due to multiple infections. HPV 16 and 18 accounted for more than 70% of the cases, while the remaining were due to the other HPV types, with HPV 45 constituting a major proportion. In squamous cell carcinomas, single infections due to HPV 16 and 18 occurred in 70% of cases, while in adenocarcinomas these two types occurred in 85% of cases. Of particular note is the high proportion of squamous cell carcinomas (10.9%) due to HPV 45. Almost all infections were high risk HPV types, with exception of two low risk HPV (44 and 70) which were seen as part of multiple infections. There were only 5 samples classified as HPV X (HPV/DNA positive and genotype negative); we hope to be able to genotype these samples in the future. HPV 16 and 18 were the 2 most common types identified throughout the study period indicating viral genotype stability in an era in which HIV started to emerge.

The prevalence of HPV DNA in cervical cancer varies in many studies, with some investigators reporting almost 100% [2,4]. This is probably attributable to the efficient extraction of intact DNA from the fresh cervical scrapes or cervical tissues used in these studies. The prevalence of HPV positive samples in our study is relatively low, but higher than that found in cervical cancer cases from Mauritius [15]. Similar low detection rates of HPV in archival cervical tissue have been reported recently from Norway [16].

In our study, all the HPV/DNA negative samples were tested for β-globin gene. All were found to be β-globin gene negative, although the β-globin gene primer used was slightly longer than the very short primer used in the DEIA reaction. Thus, the low detection rate is very likely due to poor quality of the DNA in some archival tissue samples. It has been demonstrated that suboptimal processing of specimens in the histological process such as the use of unbuffered formalin as a fixative, which was common before 1970, may yield DNA that is unsuitable for HPV detection [16]. In a subset of 200 samples from the large international RIS HPV TT study, the HPV negative samples were further explored and 89% of them were found to be β-globin gene negative, thus attributing this relatively high rate of HPV negativity to the poor quality of these retrospective samples. It was also found that adenocarcinomas were more likely to be HPV negative than squamous cell carcinomas [17].

HPV 16 and 18 are the major aetiological risk factors for cervical carcinoma. Our overall prevalence of the two major HPV types (16 and 18) was over 70%, which is similar to the IARC multicentric case-control study by Castellsague et al. [1], the study by Hammouda et al. from Algeria [18] that both used fresh samples and GP5+/6+ PCR assay, as well as the most recently meta analysis of the published literature [9,10]. The study of Castellsague et al. [1], which provides pooled data of eight case control studies conducted in North Africa (Algeria, Morocco), South America (Brazil, Paraguay, Peru) and South East Asia (India, Thailand, Philippines), showed HPV 16 prevalence of cervical adenocarcinomas of 42.7%, HPV 18 at 31.8% and HPV45 3.8% [1]. However, regional differences were noticeable with a high prevalence of HPV 16 in North Africa and South America, while in South East Asia there was a high prevalence of HPV 18. A recent study from Mozambique [19] on invasive cervical cancer reported a similar prevalence of HPV 16 (about 47%) and of HPV 16 and 18 combined (about 71%) as in our study. Compared with the results of pooled analyses of invasive cervical cancer from different continents [9,10,20], our study presents a lower prevalence of HPV 16 (44.7%) than in Europe, America, Northern Africa and South Asia but comparable with Sub-Saharan Africa, while the HPV 18 prevalence is higher (28.8%) than in all regions [9]. Considering the adenocarcinomas only [1], the prevalence of HPV 16 in our study is lower (35.0%) than in North Africa (72.7%), South America (71.7%) and comparable with the prevalence in South-East Asia (34.6%). The prevalence of HPV 18 in adenocarcinomas in our study (50%) is somewhat lower than in South East Asia, but more than twice higher than in the other regions.

The causes of the regional differences in HPV subtype prevalence need clarification, e.g. the results from Mauritius [15], where only HPV 18 was detected, warrants a follow up study. The proportion of HPV types in the two major histological types of cervical carcinoma, i.e. squamous cell carcinoma and adenocarcinoma have been noted to vary, although HPV 16 tend to predominate in squamous carcinomas and HPV 18 in adenocarcinomas. In the study of Bosch et al. [4] HPV 16 occurred in 51.2% and 28% of squamous carcinomas and adenocarcinomas respectively, while the prevalence of HPV 18 was 56% in adenocarcinomas and 12.1% in squamous carcinomas. In distinction from Bosch et al. results [4], our data shows a lower prevalence of HPV 16 in squamous cell carcinomas but a higher in adenocarcinomas, and a higher prevalence of HPV 18 in squamous tumours, lower in adenocarcinomas. We also did not observe types 31, 39 and 52 in adenocarcinomas, suggesting that these types may be less commonly associated with risk of adenocarcinomas which is in accordance with previously reported data [1]. The absence of these types was also found by other investigators [18,21] but not by the preliminary data of the RIS HPV TT study [22].

One of the interesting findings of our study was the five cases in which the samples were positive for HPV, but specific HPV genotypes could not be identified. This raises the possibility that there may be some unidentified HPV types or that some additional HPV types could have a wider range of probes. In a study in Mozambique [23], HPV specific types could not be identified in 19% of the HPV positive cases, and this failure was attributed to the limited number of HPV specific probes used.

Multiple infections were seen in only four HPV positive cases (3.5%) in this study. This is similar to some previous studies where the prevalence varied from 2.5% in Spain and Columbia [5], 3.9% in Thailand [6], 4.3% in Brazil [24], 7.9% in Morocco [25], and 12.9% in women with squamous tumour in Peru [26]. However, much higher rates were also reported by other workers, from 19.3% in Paraguay [27], 32.2% in Korea [28], 32% in Costa Rica [29] and 34.3% in Mozambique [30]. These differences could be due to underlying prevalence of multiple infections in the various populations or to differences in the methods used in detection and diagnosis [31].

Whether multiple HPV infections are involved in cervical cancer pathogenesis is rather unclear. Herrero et al. found that the risk of cervical cancer associated with HPV 16 alone is similar or greater than the risk associated with multiple infections (HPV 16 plus other HPV) [29]. The results of Lee et al. found multiple infections associated with increased cervical cancer risk [32]. Although the number of cases in our study is rather small, the results have some important implications. If the HPV vaccines under trial containing 16 and 18 antigens are close to 100% efficacy [11,13], then one could assume that about 70% of cervical cases would be prevented by the use of the vaccine. It also raises the question whether other HPV types should be included in future HPV vaccines. This is of concern because of multiple infections or infection with other common HPV genotypes which could account for 5% to 10% of cervical cancer cases. The results of our study are in agreement with previous reports by Hammouda et al. [18], and Castellsague et al. [1].

Our study had a number of strengths. The method we used is sensitive and has high efficacy in comparison with other methods using other general HPV primer sets [14]. In addition, extreme precautions were undertaken to avoid contamination, and stringent SOP (standard of procedures) were applied throughout the study. To avoid false positive results, controls consisting of non-tumour tissue blank paraffin sections and other negative control samples were simultaneously tested in each of the different steps of analysis.

One limitation of our study was the relatively small number of cases tested. Since the specimens came from different parts of the country, the duration of fixation were not uniform and could have contributed to some cases being negative for HPV. In addition, the use of non buffered formalin could also have deteriorated the DNA, resulting in false negative results.

In our study we did not have information on HIV serology. HIV infection is a risk factor for pre cervical cancer lesions [33,34] and also probably for invasive cervical cancer [34], although some earlier studies did not find any associations [35]. Patients with HIV seem to have infections with a broader spectrum of HPV types other than HPV 16, and more prone to simultaneous multiple HPV infections. The prevalence of HIV in Uganda could have been as high as 20% during part of the time when the samples were collected. This is based on HIV seroprevalance among pregnant women which rose from 10% in 1985 to around 30% in 1990–1992 [36]. Although this is a very high rate of HIV, we did not detect in our samples a different HPV genotype distribution of the most frequent types, neither a higher number of multiple infections.

## Conclusion

The results of our study show that the HPV type distribution can be determined using existing archived biological materials, such as paraffin blocks, allowing evaluating changes over time, and reaffirm the role of HPV in cervical cancer pathogenesis in the Ugandan population. HPV 16 and 18 account for about 75% of cases while about 20% are due to other HPV genotypes, and the remaining due to multiple HPV types. The results suggest that more HPV types are yet be identified and recommend that future HPV vaccines be tailored according to local HPV type distribution.

## Abbreviations

CIN: Cervical Intraepithelial Neoplasia; DDL: Delft Diagnostic Laboratories; DEIA: DNA Enzyme Immunoassay; DNA: Deoxyribonucleic acid; H&E: Haematoxilin and Eosin; HIV: Human Immunodeficiency Virus; HPV: Human papillomavirus; ICO: Institut Catala d'Oncologia; LiPA: Line Probe Assay; OD: Optical density; PCR: Polymerase Chain Reaction; RIS HPV TT: Retrospective International Study on HPV Distribution among Cases of Invasive Cervical Cancer; SOP: Standard of procedures; SPF 10-PCR: Short-Fragment PCR; VLPs: Virus-like particles.

## Competing interests

All authors declare no competing interests in regard to any financial or non-financial relationship.

The founding institutions had no influence on study design, data collection, analysis, interpretation, writing report and decision to submit the paper for publication.

## Authors' contributions

MO, EW and SdS were responsible for the concept drafting, full proposal development and getting approval from the ethics committees. MO was responsible for the specimens selection and data file cleaning. JK and WQ were responsible for the HPV DNA detection and quality control analysis. MO, EW, SdS wrote the manuscript and EW and MO were responsible for the preparation of the manuscript for submission. XFB planned the international collaborative project on HPV in invasive cervical cancer, from which Uganda is one collaborative centre.

## Pre-publication history

The pre-publication history for this paper can be accessed here:


